# Large language model-assisted natural language processing reveals stigmatizing discourse regarding psoriasis on social media

**DOI:** 10.1016/j.jdin.2025.10.005

**Published:** 2025-10-21

**Authors:** Zijun Wu, Zhuohao Song, Shiqi Zhang, Shenxin Li, Yi Xiao, Minxue Shen

**Affiliations:** a Department of Social Medicine and Health Management, Xiangya School of Public Health, Central South University, Changsha, China; b Department of Surveying and Remote Sensing Science, School of Geosciences and Info-Physics, Central South University, Changsha, China; c Department of Computer Science, School of Computer Science and Engineer, Central South University, Changsha, China; d Department of Dermatology, Xiangya Hospital, Central South University, Changsha, China; e Division of Public Health and Disease Cohort, Furong Laboratory, Changsha, China

**Keywords:** digital health, large language model, NLP, psoriasis, social media, stigmatization

*To the Editor:* Psoriasis is a chronic inflammatory skin disease with visible manifestations that result in social stigmatization and psychological distress.^1^^,^^2^ Despite the therapeutic revolution achieved by interleukin 17/23 inhibitors, misconceptions remain deeply entrenched. Stigmatization remains a core psychosocial burden for individuals with psoriasis worldwide.^3^^,^^4^

In February 2025, a high-profile incident occurred in Shanghai: a patient with psoriasis was refused accommodation, his used linen destroyed, and his condition disclosed in a hotel industry chat group as a “severe contagious skin disease.” Related videos on Douyin (Chinese TikTok) attracted >3.36 million views, generating widespread public debate.

We applied an artificial intelligence-based natural language processing framework (DeepSeek-V3) to analyze 1677 valid comments from the top 3 related videos. Comments were classified into stigmatization, destigmatization, or neutral categories according to 26 predefined rules. A 10% manually (168 comments) annotated sample was used as gold standard validation.

Stigmatization dominated online discourse, comprising 55.1% (95% confidence interval [CI], 52.7-57.5) of comments (924/1677) and attracting the greatest endorsement (47.0% like ratio) (Fig 1). Destigmatization was scarce (11.5% [95% CI, 10.0-13.0]), mainly correcting misconceptions about contagion, whereas neutral content accounted for 33.4% (95% CI, 31.2-35.7). Indirect stigma—such as moral justification of hotel actions—accounted for 71.7% of stigmatization, indicating that discrimination was frequently framed as “reasonable practice” rather than hostility.

**Fig 1:**
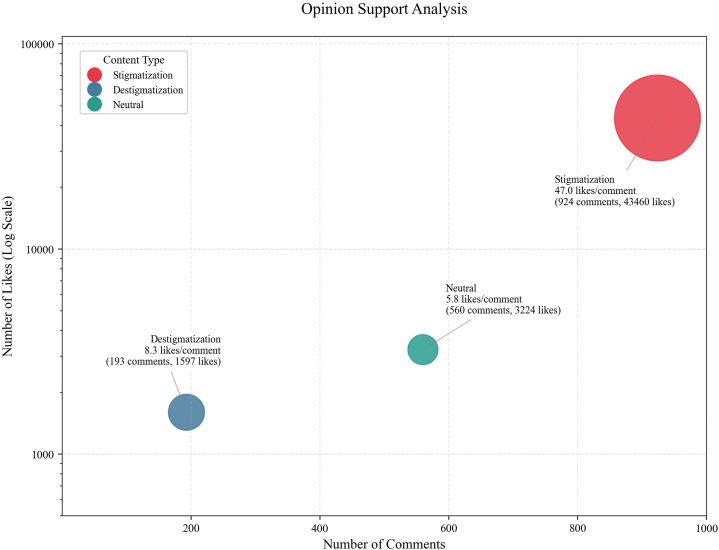
Bubble plot of opinion support analysis in comments. The y-axis represents likes counts, the x-axis indicates comment counts per category. Bubble size reflects the likes-to-comment ratio, with larger bubbles denoting higher public support for the opinion category.

Keyword analysis (Fig 2) confirmed this pattern: stigmatization manifested in multiple forms, including indirect endorsement of discriminatory actions and direct expressions such as “disgusting” or “aversion.” In contrast, destigmatizing remarks primarily focused on explaining psoriasis’ genetic basis and noncontagious nature. This asymmetry—multidimensional forms of stigmatization confronting a singular, biomedical explanation—constitutes an anguished social outcry, although many patients lack the opportunity to voice their experiences.

**Fig 2:**
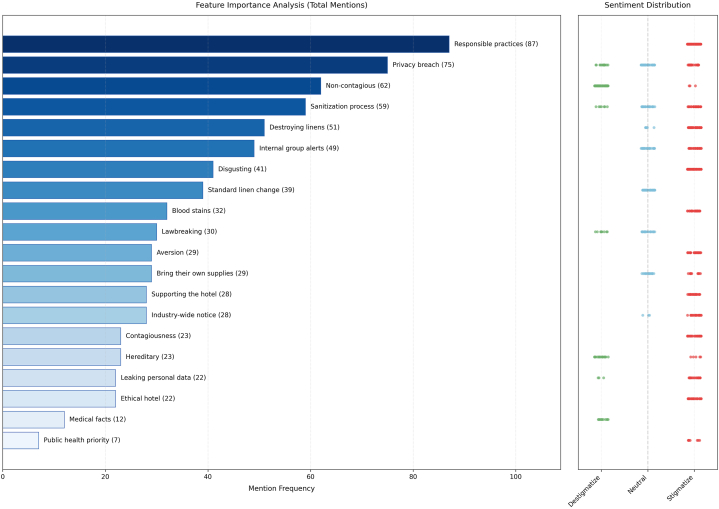
Frequency distribution of comment categories by top keywords. The left panel displays the top 20 most frequent classification keywords (bar height = frequency). The right panel shows the final classification distribution of comments containing each keyword.

Validation demonstrated robust model performance, particularly for destigmatization (precision 1.00 [95% CI, 1.00-1.00] and sensitivity 0.85 [95% CI, 0.70-0.97]). Misclassification patterns, particularly the frequent assignment of stigmatization comments to neutral, revealed cognitive dissonance between disease control and patient rights, and showed how metaphorical “benevolent prejudice” normalizes exclusion as common sense.

Our findings highlight a striking gap between medical progress and social perception. Even as biologics achieve near-complete skin clearance, many patients continue to endure social exclusion, emotional distress, and diminished quality of life. Stigmatization has been linked to higher risks of depression and suicide, reinforcing its relevance as a public health priority rather than a cosmetic concern.

The persistence of stigma beyond cured skin underscores the need for dermatologists and public health professionals to integrate stigma awareness into care. Effective strategies must address both clinical and social dimensions—incorporating stigma assessment into patient management, strengthening digital governance to curb amplification of discriminatory content, and promoting public education that emphasizes dignity and equality, not merely noncontagion.

This study demonstrates the utility of artificial intelligence-assisted discourse analysis in capturing nuanced stigmatization patterns at scale. Although conducted in China, the findings resonate globally, as stigma similarly affects patients with other visible skin diseases such as vitiligo or hidradenitis suppurativa. Bridging this gap requires a syndemic perspective that links biomedical advances with psychosocial recovery, ensuring that therapeutic success translates into genuine improvements in patients’ lived experiences.^5^

Supplementary material available on Mendeley at https://data.mendeley.com/datasets/9xxyv36zdn/1

## Conflicts of interest

None disclosed.
